# Decade of the Mind

**DOI:** 10.1186/1747-5341-3-7

**Published:** 2008-02-20

**Authors:** Manfred Spitzer

**Affiliations:** 1 Department of Psychiatry, University of Ulm, Germany; 2 Psychiatric Hospital at the University of Ulm, Director of the Transfer Center for Neuroscience and Learning (ZNL), Beim Alten Fritz 2, D-89075, Ulm, Germany

## Abstract

In the Fall of 2007, ten neuroscientists published a proposal for an interdisciplinary research initiative, the *Decade of the Mind*, that would focus on four "broad but intertwined areas": mental health, research on high-level cognitive functions, education, and computational applications (such as intelligent machines). I review the basic ideas behind the proposal and discuss the four proposed areas of research. I argue that for research on higher cognitive functions and in particular, for research and practice in education, the *Decade of the Mind *is a welcome initiative that may change our lives for the better. Therefore, the proposal, which is scientifically interdisciplinary in nature, has to be politically international.

## 

On May 21st and 22nd 2007, the Krasnow Institute for Advanced Study at George Mason University in Fairfax, VA, hosted a Symposium entitled *Decade of the Mind*. Its mission statement was later published as a letter in *Science *magazine [1]. What was proposed? Why was it proposed? Why now, and does this make sense? Ten eminent neuroscientists signed the letter in *Science *calling for an interdisciplinary research initiative "across disparate fields such as cognitive science, medicine, neuroscience, psychology, mathematics, engineering, and computer science." The authors propose that this research initiative should focus on four areas, i.e., mental health, research on high-level cognitive functions, education, and computational applications (such as intelligent machines). The agenda proposed is based upon the assumption that "such an understanding will have a revolutionary impact on national interests in science, medicine, economic growth, security, and well-being," and, in consequence, "improve our lives and our children's lives." And why now? Because "a deep scientific understanding of how the mind perceives, thinks, and acts is within our grasp."

We are left with the question: Does this make sense? Let me state upfront that I could not more agree with the ideas and the good intent of the authors, some of whom I know personally. After a *"Decade of the Brain" *[2], a *"Decade of the Mind" *makes sense because when we think of brain functions, we think of perception, motor control, thought and action. We hardly think of trust [3], love [4], or gratitude [5], let alone the stock market [6], the justice system [7], schools [8], or social norm compliance [9]. But these are all domains where neuroscience not only makes progress, but also breaks through traditional boundaries between the sciences and the humanities. In a way, one may say that after "brain-functions" such as perception and motor control had been extensively studied during the decade of the brain, investigators in systems neuroscience turned their attention and a powerful arsenal of methods towards what traditionally were regarded as "mind-functions." A *Decade of the Brain *has quite naturally led to a *Decade of the Mind." *Consequently, a *Decade of the Mind *initiative can highlight, consolidate, and dramatically advance exciting scientific progress as profound as any in fields which traditionally receive most attention, such as cosmology, particle physics and molecular biology.

An example follows from mental health. In the 1970s, when I was a young resident in psychiatry at a German University Medical Center, colleagues from the neighboring psychotherapy training program and clinic sometimes presented me with the following challenge: They could, they informed me, by talking through the problems and utilizing psychoanalytic techniques to probe the innermost workings of the mind deep down, treat an intellectually capable depressed patient with skillful conversations. At the time back in Germany, such treatment was referred to as "Tiefenpsychology" ("deep psychology"). In contrast, we psychiatrists might treat an equally depressed patient, typically a patient not as young, attractive, verbal, intelligent or successful (the so-called "YAVIS" patient) with little more than an antidepressant medication. Was not this latter approach a superficial one, my depth-psychology colleagues asked me, was I not chemically addressing some symptoms, without provide a "real cure"? And even though we often saw the medicated patient getting better within a few weeks, albeit sometimes with medication-related side effects, while the articulate patient sometimes remained on the couch for years, we nonetheless felt awkward.

Twenty-five years on, this situation can be framed intriguingly differently. Antidepressant medications may promote growth of new neurons [10,11] at exactly the location where neurons die due to increased stress [12]. So we may begin to understand how stress causes mental dysfunction, and how medications might work by restoring the hardware necessary to function properly. And "talk therapy?" Emerging insights from affective neuroscience continue to maintain focus on the social dimensions of the brain [13,14]. Deeply? We have much to learn: On the one side, it is not clear if neurogenesis in the hippocampus is the cause of the antidepressant effect or simply correlated with it. On the other, talking to an ill patient may be akin to typing into a word processor "don't crash" after your computer has just crashed. Word processing does not reach the inner workings of the CPU in order to correct a problem deep down in the system. Recent empirical research on the unconscious workings of our mind has revealed that "talking" may in fact be a quite superficial approach to brain function [5]. At least for some forms of depression, "the talking cure" is perhaps superficial while the drugs work on the cause of the disorder. This would be what used to be – upside down!

We did not need a *Decade of the Mind *to bring about expand vistas as described above. The ultimate reason is that for about a century and a half the medical profession has seen itself as applied science and hence will enthusiastically embrace and use insights from basic research for clinical applications. Necessary clinical research structures – transferring a newly discovered biochemical pathway, receptor, ligand, gene (or what have you) – are in place and slowly but securely sort out ideas that work from ideas that don't. Quite often, headline-making basic science discoveries do not easily make it into new and better therapies. For example, the gene for Huntington's disease was discovered in 1983 and the gene product, the protein huntingtin, was disclosed 10 years later, but there are no therapeutic consequence so far [15-17]. Despite the drama of stem cell research and its promise of customized replacement medicine, we are not there yet. And moreover, as knowledge increases, the more distant this goal appears. Progress in clinical applications is often achieved in a piecemeal fashion, with survival rates for cancer patients inching upwards slowly, following the introduction and careful testing of new combinations of new and old compounds in large-scale clinical trials. These are financed at high risk, gain, and cost, but the potential benefits drive the process quite well. As regards this type of progress, an additional initiative does not appear to be needed.

However, there will soon be more to psychopharmacology than some drop of symptoms on rating scales. For example, consider memory consolidation, reconsolidation and the treatment of PTSD. We know that newly learned information needs to be reprocessed after learning in order to be stored permanently in long term memory [18]. In fact, recent research from our laboratory suggests that the activity of hippocampal and parahippocampal areas during post-learning reprocessing is highly predictive of successful recall at a later point in time (Sokolov A, Maier C, Spitzer M, Grön G: Encoding, early consolidation, and retrieval of associative declarative memories in the waking human brain, in preparation). We further know that the activation of memory traces renders them labile, such that they need to be consolidated again after memorizing, a process called reconsolidation [19-21]. This implies that it may be possible to talk through a problematic experience with a PTSD patient; administer a drug that dampens, or completely blocks, reconsolidation; and thereby erase the memory traces that haunt the patient. Research on such integrated psycho-pharmaco-therapy is unlikely to be sponsored by the drug-industry, just as they hardly invest in systems neuroscience level investigations of mental disorders (but rather narrowly focus upon the genetic and molecular level of inquiry). At this crucial junction of pharmaco- and psychotherapy, the *Decade of the Mind *initiative may well help the push for new knowledge.

Robotics and intelligent machines are another field mentioned by the proponents of the *Decade of the Mind*. Research on neural network modeling of brain style pattern recognition, on artificial life with evolving algorithms, on agents and distributed control, and on information technology have led to changes in our lives that were hardly imagined two decades ago. As Moore's law still holds (with doubling performance and halving prices of information technology products at the breathtaking speed of 18 months), and as the fruits of this work shows up everywhere and as information technology, even after the burst of the stock bubble in 2000, is still the horse pulling Western economies as well as the cow milked by them, there appears little need for public money to drive this process even faster.

So, of the four arenas of the *Decade of the Mind*, two remain: research in high-level cognitive (i.e., "mindful") functions and education. In my view, these are the most two critically valid domains of the *Decade of the Mind*.

One of the motors of the *Decade of the Brain *was the advent of functional magnetic resonance imaging in 1991. Whereas in the beginning this was used to study rather low-level mental processes, such as visual perception of flickering light [22] and finger tapping [23], fMRI is now used for the exploration of any function of the mind that a researcher can imagine. As mentioned above, social behaviors, trust, love, meditation, and prayer have been studied. The fledgling new area of neuroeconomics [24] is in part driven by the cooperation of economists and neuroscientists studying complex social behavior in the scanner. Such "social neuropsychology", or *social neuroscience*, as it is now called, was unthinkable a decade ago, and is likely to transform our understanding of the very nature of human beings. In fact, there is now a chance that the scientific achievements and conceptual advances will finally enable scientists and humanists to surpass the science-humanities divide [25] that has plagued discourse and progress concerning human nature for several decades. Synergy of this sort is urgently needed, it seems to me, if human beings are to have a chance to survive this century.

Finally, the most important achievement of the *Decade of the Mind *may be the idea that neuroscience is to education as biochemistry and genetics are to medicine or physics is to architecture. Paradoxically, just as two editorials on neuroscience and education in Science magazine over the past two years were skeptical, hesitant, and unimaginative [26,27], the proponents of the *Decade of the Mind *are strikingly cautious on this topic and merely mention it in passing. But think about it: The primary task of the brain is learning. Brain researchers produce an annual output of about 40,000 scientific papers [28]. So is it really possible, as the writer of one of the editorials firmly states [26], that neuroscience has nothing to say about schools? Notably, neither editorial was written by neuroscientists, and both devoted most of the text to caution against simplified neuro-myths. While I could not agree more – "brain-based learning" is just as trivial as "leg-based running" – they do not provide us with any new insight on the positive side, i.e., as to what we should actually do. Too bad that the *Decade of the Mind *initiative is not more upbeat in this respect. In order to really begin to tackle the problem of how to best educate and teach children, a *vision *is needed of what to do in order to progress in the field of brain research and education.

In my view, a lot can be learned in this respect from the history of medicine. 200 years ago, medicine was little more than a mixture of bits of knowledge, fads and plain quackery. In the middle of the nineteenth century, Hermann von Helmholtz, Ernst Wilhelm von Brücke, Emil Du Bois-Reymond and a few other great minds got together and drew up a grand scheme for what medicine should be: applied natural science [29]. This happened at a time when – from our perspective – very little was known: cellular pathology, microbiology and pharmacology hardly existed as domains of scientific investigation, let alone as tools for physicians. But the idea – medicine is applied science – caught on and led to improvements so dramatic that today we can hardly pay for them but nonetheless want them for everybody. The progress was *not *made by the clinician asking a biochemist at the bedside: "Now, aren't you claiming that all that goes on in the body is biochemistry. If that's so, how to fix this one?" It took the advent of statistics and a huge amount of the above mentioned applied clinical research with carefully conducted large scale trials that compare different methods of treatment against each other. These studies are *informed *by biochemistry so that we do not compare the effects of *any *drug in *any *illness (this would be ridiculous and not feasible), we rather form hypotheses drawn from insights into mechanisms and then test them in the field.

This is precisely what we must do in order to make progress in education. "You claim all learning is taking place in the brain. If that's so, which type of preschool is most effective? – From a medical perspective, it is obvious that a neuroscientist cannot answer such questions sometimes posed by educators or educational policy makers [27]. But it is just as clear that the answers will come from research *informed *by developmental cognitive neuroscience [30-32]. I agree wholeheartedly with the proponents of the decade of the mind that we need what they call "translational research", i.e., a dialogue and applied research, in order to make real progress in evidence based education.

This is by no means trivial. As every department head of a hospital with a strong research orientation knows, there is always the tendency for the institution to degenerate into a city-hospital with a lab in the basement and no relationship between the two. Laboratory based researchers tend to disregard clinical work as "messy", and clinicians tend to think of basic science as theoretical and hence unrelated to their practice. Just as it is the job of the department head to constantly encourage and implement mutual contact between the members of these two cultures (sometimes resembling more two quite different species) in order to make basic work meaningful (ask the right questions in the lab) and clinical work informed (bring the right conceptual framework to the bedside), it takes great effort to not just talk about, but really carry out "interdisciplinary" or "translational" investigations. Given the urgency of educational improvements across the globe, it is frustrating to find translational research ridiculed in the very issue of Science magazine that contained the proposal for a *Decade of the Mind *initiative [33]. The main purpose of the brain/mind is to learn and to use the stored information for survival. If mankind is to survive, we need to take the world's youngest citizens and their education seriously and *do *the necessary research, modeled on the vision and success of medicine. Yes, we need a *Decade of the Mind *and we welcome, within this decade, education and learning as a primary focus.

One final very important remark: The call for a *Decade of the Mind *in Science magazine by ten USA scientists seeks a project with explicitely "*national *(emphasis mine) interests in science, medicine, economic growth, security, and well-being." Emphatically, the *Decade of the Mind *must be a global initiative, and certainly not merely a USA initiative concerning *national *(USA) science and medicine, the *national *(USA) economy, and *national *(USA) security and well-being. Just as global warming affects all of us and needs to be studied and dealt with on a global scale, the mind is something that should be studied from all angles of the globe and within all cultural backgrounds and contexts. Let's not waste time and let's ALL get started! All of humankind!

## Competing interests

The author(s) declare that they have no competing interests.

## References

[B1] Albus JS, Bekey GA, Holland JH, Kanwisher NG, Krichmar JL, Mishkin M, Modha DS, Raichle ME, Shepherd GM, Tononiet G (2007). A proposal for a decade of the mind initiative. Science.

[B2] Bush GHW (1990). Proclamation "Decade of the brain," 1990–2000. Proclamation 6158" Federal Register 55.

[B3] King-Casas B, Tomlin D, Anen Cedric, Camerer CF, Quartz SR, Montague R (2005). Getting to know you: Reputation and trust in a two-person economic exchange. Science.

[B4] Bartels A, Zeki S (2004). The neural correlates of maternal and romantic love. NeuroImage.

[B5] Haidt J (2005). The Happiness Hypothesis Finding Modern Truth in Ancient Wisdom.

[B6] Kuhnen CM, Knutson B (2005). The neural basis of financial risk taking. Neuron.

[B7] Zeki S, Goodenough O (2006). Law and the Brain.

[B8] Spitzer M On Learning Brain Research and the School of Life.

[B9] Spitzer M, Fischbacher U, Herrnberger B, Grön G, Fehr E (2007). The neural signature of social norm compliance. Neuron.

[B10] Santarelli L, Saxe M, Gross C, Surget A, Battaglia F, Dulawa S, Weisstaub N, Lee J, Duman R, Arancio O, Belzung C, Hen R (2003). Requirement of hippocampal neurogenesis for the behavioral effects of antidepressants. Science.

[B11] Sahay A, Hen R (2007). Adult hippocampal neurogenesis in depression. Nat Neurosci.

[B12] Sapolsky R (1992). Stress, the Aging Brain, and the Mechanisms of Neuron Death.

[B13] Panksepp J (1998). Affective Neuroscience: The Foundations of Human and Animal Emotions.

[B14] Schmidt L (2003). Special issue on affective neuroscience. Brain and Cognition.

[B15] Gusella JF, Wexler NS, Coneally PM, Naylor SL, Anderson MA, Tanzi RE, Watkins PC, Ottina K, Wallace MR, Sakaguchi AY, Young AB, Shoulson I, Bonilla E, Martin JB (1983). A polymorphic DANN marker genetically linked to Huntington's disease. Nature.

[B16] McDonald ME, Ambrose CM, Duyao MP, Myers RH, Lin C, Srinidhil L, Barnes G, Taylor SA, James M, Groot N, McFarlane H, Jenkins B, Anderson MA, Wexler NS, Gusella JF, Bates GP, Baxendale S, Hummerich H, Kirby S, North M, Youngmans S, Mott R, Zehetner G, Sedlacek Z, Poustka A, Frischauf AM, Lehrach H, Buckler AJ, Church D, Doucettestamm L (1993). A novel gene containing a trinucleotide repeat that is expanded and unstable on huntingtons-disease chromosomes. Cell.

[B17] DiFiglia M, Sapp E, Chase K, Schwarz C, Meloni A, Young C, Martin E, Vonsattel J-P, Carraway R, Reeves SA, Boyce FM, Aroni N (1995). Huntingtin is a cytoplasmatic protein associated with vesicles in human and rat brain neurons. Neuron.

[B18] Lechner HA, Squire LR, Byrne JH (1999). 100 years of consolidation – Remembering Müller and Pilzecker. Learning and Memory.

[B19] Myers KM, Davis M (2002). Systems-level reconsolidation: Reengagement of the hippocampus with memory reactivation. Neuron.

[B20] Nader K, Schafe GE, LeDoux JE (2000). Fear memories require protein synthesis in the amygdala for reconsolidation after retrieval. Nature.

[B21] Debiec J, LeDoux JE, Nader K (2002). Cellular and systems reconsolidation in the hippocampus. Neuron.

[B22] Belliveau JW, Kennedy DN, McKinstry RC, Buchbinder BR, Weisskoff RM, Cohen MS, Vevea JM, Brady TJ, Rosen BR (1991). Functional mapping of the human visual cortex by magnetic resonance imaging. Science.

[B23] Kim SG, Ashe J, Hendrich K, Ellermann JM, Merkle H, Ugurbil K, Georgopoulos AP (1993). Functional magnetic resonance imaging of motor cortex: Hemispheric asymmetry and handedness. Science.

[B24] Spitzer M (2006). Neuroeconomics: Values and decisions in the brain. Neuropsychoeconomics.

[B25] Snow CP (1993). The two cultures.

[B26] Stern E (2005). Pedagogy meets neuroscience. Science.

[B27] Hirsh-Pasek K, Bruer JT (2007). The brain/education barrier. Science.

[B28] Anonymous (1999). Celebrating a decade of progress (Editorial). Nature Neuroscience.

[B29] Sulloway F (1979). Freud Biologist of the mind.

[B30] Gros-Louis JG, West MJ, Goldstein MH, King AP (2006). Mothers provide differential feedback to infants' prelinguistic sounds. International Journal of Behavioral Development.

[B31] Saffran JR, Thiessen ED, Hoff E, Shatz M (2007). Domain-general learning capacities. Handbook of Language Development.

[B32] Donovan W, Leavitt L, Taylor N, Jennifer Broder J (2007). Maternal sensory sensitivity and response bias in detecting change in infant facial expressions. Infant behavior and development.

[B33] Greenberg DS (2007). On the road to academic greatness – a parable. Science.

